# Ultra-Sensitive Nanofiber Fluorescence Detection in a Microfluidic Chip

**DOI:** 10.3390/s150304890

**Published:** 2015-02-26

**Authors:** Zhiyong Li, Yingxin Xu, Wei Fang, Limin Tong, Lei Zhang

**Affiliations:** State Key Laboratory of Modern Optical Instrumentation, Department of Optical Engineering, Zhejiang University, Hangzhou 310027, China; E-Mails: lizhiyong@zju.edu.cn (Z.L.); noultimate@zju.edu.cn (Y.X.); wfang08@zju.edu.cn (W.F.); phytong@zju.edu.cn (L.T.)

**Keywords:** micro/nanofiber, biconical taper, microfluidic chip, fluorescence sensor

## Abstract

We report an ultra-sensitive and robust fluorescence sensor made by using a biconical taper with a waist diameter of 720 nm for both excitation and fluorescence collection. To enhance the stability of the fluorescence sensor, the biconical taper has been embedded in a 125 µm wide microchannel with a detection length of 2.5 cm. Investigated by measuring the fluorescence intensity of rhodamine 6G (R6G), the sensor shows a detection limit down to 100 pM, with excellent reversibility in a concentration range of 0–10 nM. The sensor has also been applied to quantum dot (QD)-labeled streptavidin measurements, yielding a detection sensitivity down to 10 pM for QDs. In addition, the small sample volume (*ca.* 500 nL), high sampling throughput, and seamless connection between the biconical taper and standard optical fibers offer a number of attractive advantages for chemical and biosensing applications.

## 1. Introduction

Optical microfibers and nanofibers (MNFs) have been emerging as a novel platform for exploring fiber-optic technology on the micro/nanoscale owing to their outstanding properties including low waveguiding losses, tight optical confinement, strong evanescent fields, and widely tailorable waveguide dispersion [1,2,3,4]. Among various MNFs applications, optical sensing has been attracting increasing research interest due to its possibilities of realizing miniaturized fiber optic sensors with small footprint, high sensitivity, fast response, high flexibility and low optical power consumption [5]. Typical MNFs sensing structures include biconical tapers [6,7], optical gratings [8,9], circular cavities [10,11,12], Mach-Zehnder interferometers [13,14,15] and functionally coated/doped MNFs [16,17]. Based on biconical tapers, evanescent wave spectroscopy is a powerful method for obtaining the concentration and structure information of liquid or solid samples via absorption [18,19] and fluorescence [20,21] measurements.

The evanescent wave fluorescence sensor is a well-developed tool for a wide range of applications, particularly for biological sensing [22,23], since fluorescence photons can be efficiently coupled into guided modes of optical nanofibers [24,25,26], which offers a possibility for single molecule or single nanoparticle sensing. In the last decade a number of biconical taper-based fluorescence biosensors have been developed. These sensors are capable of detecting with ease fluorescence from solutions or particles/molecules deposited on the fiber. For example, Wiejata *et al.* experimentally demonstrated a fluorescence sensor by using a biconical taper with a waist diameter of 3.69 µm, and a waist length of 7.1 mm. Flourescein solution at concentrations of 10 to 60 µM were placed in the waist region, and excitation light of 460 nm was launched at one end of the fiber, and 516 nm light intensity was measured at the other end of the fiber. The 516 nm fluorescence signal was found to be proportional to the fluorescein concentration [27]. In this case, the fractional power of the fundamental modes outside the taper waist (η) was about 0.2%. To enhance the sensitivity, η should be increased because fluorescence intensity is proportional to the excitation intensity. Decreasing the taper waist diameter is an effective method, which may dramatically increase η when the diameter decreases to a certain value. However, when η increases to a critical value, the fluorescence collection efficiency will decrease. For example, Stiebeiner *et al.* presented spectroscopic measurements on 3,4,9,10-perylene-tetracarboxylic dianhydride molecules (PTCDA) at ambient conditions by using a biconical taper with a waist diameter of 320 nm, and a waist length of 1 mm for both excitation and fluorescence collection. The strong radial confinement and the pronounced evanescent field of guided light in optical nanofibers yielded favorable conditions for ultra-sensitive surface spectroscopy of molecules deposited on the fiber. It was found that surface coverages as small as 1 part per thousand of a compact monolayer still gave rise to fluorescence spectra with a good signal to noise ratio [20]. Recently, Warren-Smith *et al.* developed a model for the fluorescence sensing properties of optical nanowires and compared them quantitatively with experiments. Numerical results show that the fluorescence signal is relatively sensitive to core size for low concentration sensing due to the increased evanescent field power [21]. Note that most of the previously reported evanescent wave sensors used MNFs suspended in air or mounted in a bulky volume flow chamber, thus, surface contamination and environmental factors are likely to affect the stability of these sensors. To address these issues, embedding the MNFs in a low-index material (e.g., Teflon AF) [28,29,30] can greatly enhance the stability of the MNF sensors. However, the thickness of Teflon layer has to be well controlled. If the Teflon layer is too thick, this embedded geometry would not allow samples access to the evanescent field, making the configuration useless for optical sensing and other applications.

In our previous work, we reported an evanescent wave absorption sensor made by using a silica biconical taper with a waist diameter of 900 nm, and a waist length of 2.5 cm. The biconical taper was embedded in a 125 μm wide microchannel with a detection length of 2.5 cm [31]. Because the unstretched fiber is completely embedded in the polydimethylsiloxane (PDMS), and only the taper waist used for sensing is surrounded by chemical or biological samples filling the microchannel, this configuration not only offers excellent stability and reproducibility, but also makes effective use of the pronounced evanescent wave of the centimeter-long taper waist, resulting in an ultra-high sensitivity. Here, we report an evanescent wave fluorescence sensor by using a silica biconical taper with a taper waist of 720 nm and a PDMS microfluidic chip with the aforementioned embedded configuration. Investigated by measuring the fluorescence intensity of rhodamine 6G (R6G) and quantum dot (QD)-labeled streptavidin, the sensor shows an ultra-high sensitivity owing to the strong evanescent field outside the taper waist for fluorescence excitation and the high efficiency for fluorescence collection. Because of the use of a slot sample-vial-array configuration for sample introduction, the sampling throughput can be as high as 60 samples/hour, with excellent reversibility for low concentration samples. Compared to silica and polymer nanofiber sensors [14,32], which use an evanescent coupling method for optical waveguiding, biconical tapers can seamlessly connect with light source and detector through standard fibers, yielding a simple, robust, and sensitive sensing structure.

## 2. Experimental Section

For our experiments, we used a biconical taper for both fluorescence excitation and collection. We fabricated the biconical taper by stretching a standard single mode optical fiber (SMF-28, Corning Incorporated, Corning, NY, USA) while heating it with a hydrogen/oxygen flame [33]. In brief, a 3 cm long section of the commercial single-mode fiber was stripped of its protective plastic jacket and fixed by two fiber holders. When the fiber was heated to a softening temperature, it was drawn in the horizontal plane until the waist diameter went down to the desired value. Because the pulling speed, the pulling distance, and the flow rate of hydrogen were all well controlled by a computer, we could produce biconical tapers with a homogeneous waist diameter down to 300 nm and a typical extension of 1–10 cm. In this work, the desired diameter of the taper waist was 690 nm, which is the critical diameter for single mode operation for water-clad glass MNFs at 532 nm. Based on scanning electron microscopy (SEM) measurements, the diameter of the taper waist was about 720 nm, only 4.3% greater than the desired value.

The as-fabricated biconical taper was then embedded in a PDMS microfluidic chip with a 125 µm wide, 150 µm deep, and 5 cm long microchannel under an optical microscope. To fabricate the PDMS microfluidic chip, we used a four-step fabrication procedure described in our previous work [31]. Briefly, a SU-8 master and two 375 µm diameter capillaries were used to produce the microchannel and sample inlet/outlet channels, respectively. When the biconical taper was located in the center of the microchannel with two taper transitions just located at the sample inlet and outlet holes, uncured PDMS was carefully infused into the microchannel and cured to avoid sample leaking. Finally, two 2 cm long capillaries were inserted into the sample inlet/outlet channels, one served as sampling probe, and the other connected to a syringe, which was used to generate negative pressure for sample introduction.

A slot sample-vial-array was adopted for sample introduction because of its high sampling throughput [34]. The sample vials were produced from 0.5 mL centrifuge tubes by cutting 1.5 mm wide, 2 mm deep slots on the conical bottom of the tubes for pass-through of the sampling capillary. The slotted sample vials were horizontally fixed on a glass slide in an array, with the slot of each vial positioned horizontally to allow free passage of the sampling capillary through all the vial slots sequentially by linearly moving the glass slide along the direction of the array. The vials were filled alternately with 100 µL samples and ultrapure water. Thus, the microchannel and the surface of biconical taper can be cleaned before a new sample was introduced by linearly moving the glass slide. Samples in the slot vials were sucked into the microchannel by negative pressure generated by a syringe that connected with the sample outlet channel.

**Figure 1:**
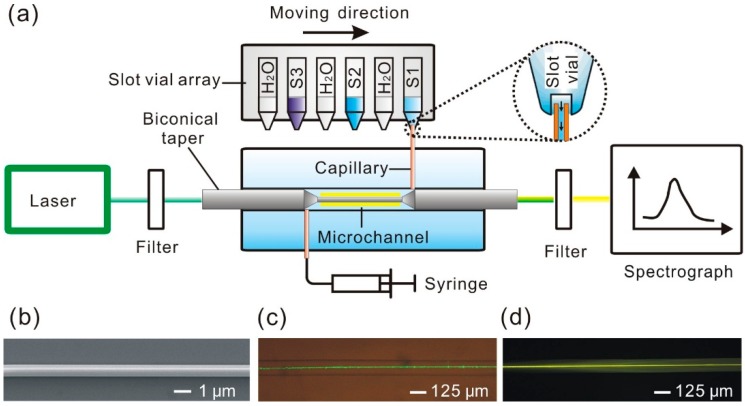
(**a**) Scheme of the experimental setup for biconical taper-based fluorescence sensing system; (**b**) Scanning electron microscopy image of a section of taper waist of 720 nm in diameter; (**c**) Optical micrograph of a 720 nm diameter taper waist guiding a laser with a wavelength of 532 nm embedded in a microchannel; (**d**) Optical micrograph of fluorescence excited by the evanescence field outside a 720 nm diameter taper waist.

Figure 1a shows the schematic experimental setup for biconical taper-based fluorescence sensing system. Excitation laser (532 nm) is coupled to the taper waist from one end of the unstretched part of the biconical taper. A fiber spectrometer (Maya 2000 Pro, Ocean Optics, Dunedin, FL, USA) is used to record the fluorescence spectra. To enhance the signal to noise ratio, a 550 nm short pass filter and a long pass filter (561 nm for R6G and 700 nm for QD-labeled steptavidin) are used to remove any scattered or excitation light from the system. As shown in Figure 1b, the as-fabricated biconical tapers exhibited a very smooth and clean surface, which is very important for low loss optical waveguiding. We have measured the transmittance of the biconical taper at 1.55 μm during the tapering process, the transmittance of the biconical taper is found to be ~95%. When ultrapure water is injected into the microchannel, the taper waist shows bright green color (see Figure 1c), indicating an evanescent field outside the taper waist. When 1 μM R6G solution is introduced into the microchannel, yellow fluorescence (see Figure 1d) is excited by the strong evanescent field. In the taper sections, the mode of the unstretched fiber is adiabatically transformed into the strongly guided mode of the ultrathin section and back, resulting in a highly efficient coupling of light into and out of the taper waist [20].

## 3. Results and Discussion

### 3.1. R6G Evanescent Field Fluorescence Measurements

**Figure 2:**
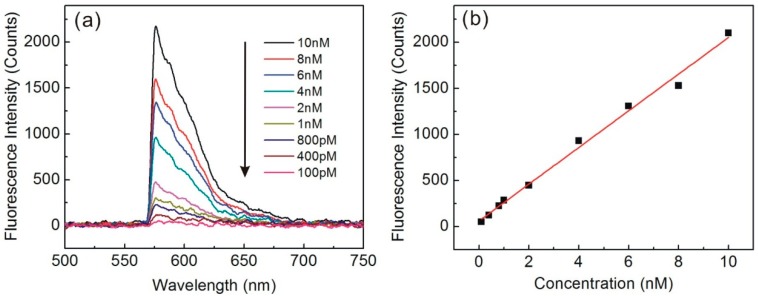
(**a**) Fluorescence spectra of R6G at different concentrations; (**b**) Fluorescence peak intensity as a function of concentration of R6G.

In this work, we systematically investigated the sensitivity and reproducibility of the sensor by measuring the fluorescence of R6G, a dye widely used in fluorescence microscopy, flow cytometry, fluorescence correlation spectroscopy and enzyme-linked immunosorbent assay (ELISA). The excitation and emission wavelength are 550 nm and 580 nm, respectively. Here, a 532 nm wavelength laser (0.6 mW) was guided into the 720 nm taper waist to excite the fluorescence. In this case, η is ~15%, which may provide a strong evanescent field for exciting the fluorescence and a high efficiency for collecting the fluorescence. Because the fluorescence collected by the biconical taper was then guided in the fiber spectrometer through a long pass filter, the fluorescence collection efficiency of the sensing system will mainly depend on the coupling efficiency of fluorescence into the guided mode of the biconical taper (~20%), the transmission of the filter (~90%), and the light collection efficiency of the spectrometer (~75%). Therefore, the estimated fluorescence collection efficiency for the sensing system is about 13.5%. Different concentrations of R6G solution ranging from 0.1 to 10 nM were prepared before use. Each sample was measured three times under the same condition, and we used the average peak intensity for the calibration curve. After each measurement, the microchannel was flushed with ultrapure water. As we can see in Figure 2a, when the R6G concentration increases, the fluorescence intensity increases obviously. Note that the fluorescence spectra do not have a Gaussian profile because a 561 nm long pass filter was used to filter out residual pump light. The average peak intensity at 580 nm wavelength versus R6G concentrations is shown in the Figure 2b. A linear response range of 0–10 nM was obtained with a regression equation of, fluorescence intensity = 199.2C + 62.3 (r^2^ = 0.992). The detection limit for R6G based on 3 times the standard deviation of the blank values was 100 pM.

**Figure 3:**
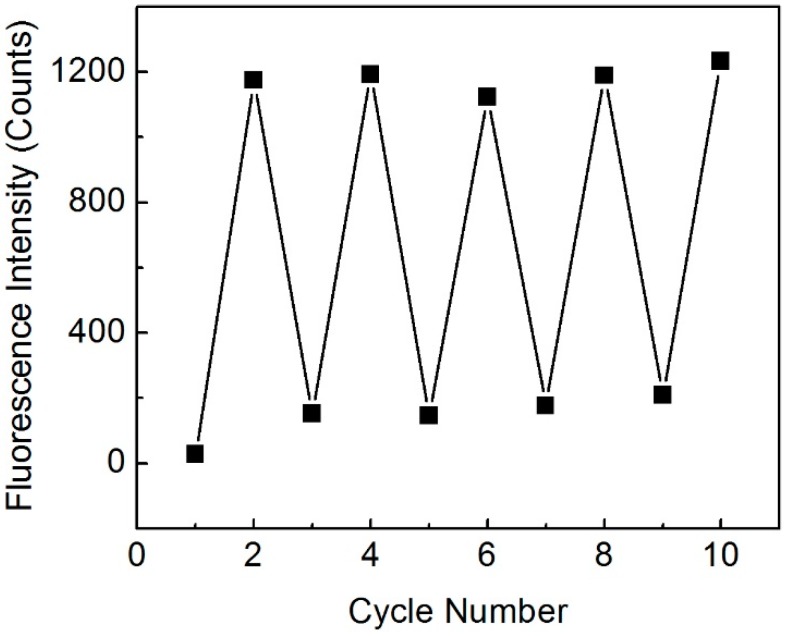
Cycling measurement of 6 nM R6G and ultrapure water.

The reproducibility of the biconical taper-based fluorescence sensing system was demonstrated in a cycling measurement of 6 nM R6G and ultrapure water under optimized conditions. A reproducibility of 3.3% RSD (*n* = 5) was achieved in the 5 cycling measurements (Figure 3). When the ultrapure water sample transmission intensity cannot reach the original level after several days’ measurements or several measurements of high concentration solutions, extensive flushing of the microchannel and the taper waist with ethanol and air alternatively will renew the taper waist surface and regain the original blank transmission intensity.

### 3.2. Quantum Dots-Labeled Steptavidin Evanescent Field Fluorescence Measurements

**Figure 4:**
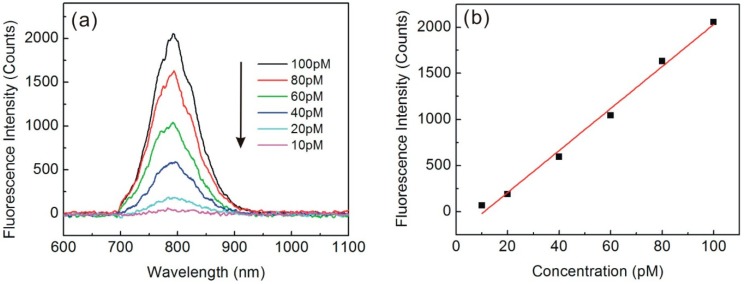
(**a**) Fluorescence spectra of QDs-labeled streptavidin at different QDs concentrations; (**b**) Fluorescence peak intensity as a function of QDs concentrations.

Compared to conventional organic fluorophores, QDs are virtually immune to the effects of photo- bleaching, and have a relatively higher absorption cross-section, which may further enhance the sensitivity. Here, we used a commercially available CdSeTe/ZnS QDs-labeled steptavidin (Wuhan Jiayuan Quantum Dots Co., Ltd., Wuhan, China) for fluorescence measurements. Each sample was measured three times under the same conditions, and we used the average peak intensity for the calibration curve. CdSeTe/ZnS QDs have been coupled to streptavidin directly through an active ester coupling reaction, which results in QDs-streptavidin conjugates with high specific biological activity. Figure 4a shows the typical fluorescence spectra of QDs labeled streptavidin at different QDs concentrations ranging from 10 to 100 pM.

The average peak intensity at 800 nm wavelength versus QDs concentrations is shown in Figure 4b. A linear response range of 10–100 pM was obtained with a regression equation of, fluorescence intensity = 22.8C − 248.7 (r^2^ = 0.991). The detection limit for QDs based on three times the standard deviation of the blank values was ~10 pM. Because there are 3–5 streptavidins covalently attached on one QD, the detection limit for streptavidin was 30–50 pM.

## 4. Conclusions

We have demonstrated an ultra-sensitive evanescent wave fluorescence sensor made by integrating a biconical taper with a microfluidic chip. The evanescent wave fluorescent sensing performance was tested by measuring R6G and QD-labeled streptavidin, achieving detection limits of 100 pM and 10 pM in terms of QDs, respectively. Also, the seamless connection between the biconical taper and standard optical fibers offers the sensor a number of attractive advantages inherited from the standard fiber optic system configuration. With recent advances in chemical and biosensing applications of MNFs, the compact and robust sensor shown here may open up new opportunities for ultra-sensitive immunoassay, ELISA, toxin measurement and detection of pathogens.
